# The Influence of Permeability through Bacterial Porins in Whole-Cell Compound Accumulation

**DOI:** 10.3390/antibiotics10060635

**Published:** 2021-05-26

**Authors:** Silvia Acosta-Gutiérrez, Igor V. Bodrenko, Matteo Ceccarelli

**Affiliations:** 1 Physical Chemistry Chemical Physics Division, Department of Chemistry, University College London, London WC1H 0AJ, UK; 2 Institute of Structural and Molecular Biology, University College London, London WC1H 0AJ, UK; 3 Institute for the Physics of Living Systems, University College London, London WC1H 0AJ, UK; 4 EPSRC/JEOL Centre for Liquid Phase Electron Microscopy, University College London, London WC1H 0AJ, UK; 5 IOM/CNR, Sezione di Cagliari, Cittadella Universitaria di Monserrato, 09042 Monserrato, Italy; bodrenko@iom.cnr.it (I.V.B.); matteo.ceccarelli@dsf.unica.it (M.C.); 6 Department of Physics, University of Cagliari, 09042 Monserrato, Italy; 7 CNR/IOM, Cittadella Universitaria di Monserrato, 09042 Monserrato, Italy

**Keywords:** Gram-negative bacteria, porins, permeability, antibiotics

## Abstract

The lack of new drugs for Gram-negative pathogens is a global threat to modern medicine. The complexity of their cell envelope, with an additional outer membrane, hinders internal accumulation and thus, the access of molecules to their targets. Our limited understanding of the molecular basis for compound influx and efflux from these pathogens is a major bottleneck for the discovery of effective antibacterial compounds. Here we analyse the correlation between the whole-cell compound accumulation of ~200 molecules and their predicted porin permeability coefficient (influx), using a recently developed scoring function. We found a strong linear relationship (74%) between the two, confirming porins key in compound uptake in Gram-negative bacteria. The analysis of this unique dataset aids to better understand the molecular descriptors behind whole-cell accumulation and molecular uptake in Gram-negative bacteria.

## 1. Introduction

From the discovery of the first antibiotics in the 1930s to the present time, antibiotics have been a significant relief for the global health burden caused by pathogenic bacteria around the globe [1]. Nevertheless, antibacterial chemotherapy has been challenged since the beginning by the appearance of antibiotic-resistant strains [2,3]. The misuse of antimicrobials in humans, animals, and agriculture [4] has accelerated antimicrobial resistance in recent decades, bringing us to the so-called ‘Resistance era’ [5]. Furthermore, because of the poor hit rates from genomics/target-led screens and high drug failure rates in late clinical development, the pipeline for the development of antibiotics is virtually empty of new scaffolds, jeopardizing modern medicine [4].

**Figure 1:**
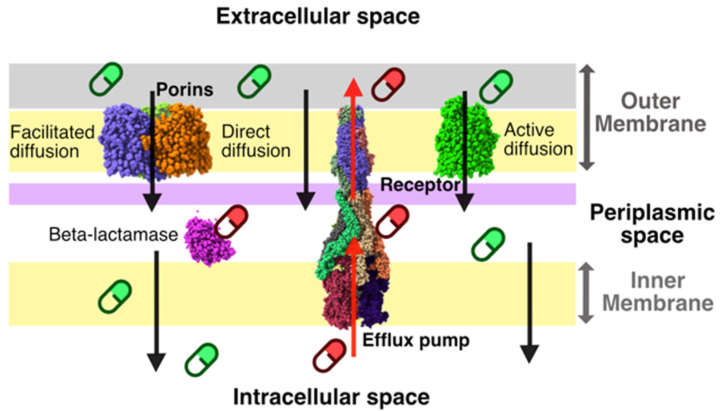
Compound accumulation process. Successful (green) versus unsuccessful (magenta) compounds.

Despite the advances in genomics and sequencing, high-throughput screening (HTS), automated chemical synthesis, and structural biology, no new classes of antibiotics against Gram-negative species [6,7,8,9,10] have been discovered. Among the ESKAPE pathogens, we find four Gram-negative species, also classified by the World Health Organization (WHO) as critical for the search of new antibiotics able to combat them [4]. All the traditional large-scale biochemical or target agnostic phenotypic antibacterial screening efforts had not been very fruitful for Gram-negative pathogens due to our limited understanding of the molecular basis for compound uptake and accumulation [11]. Their complex cell envelope, conversely to Gram-positive, comprises an outer membrane (OM) and an inner membrane (IM), which together delineate the periplasmic space [12,13] (Figure 1).

The OM creates an additional physical/mechanical barrier that protects the cell against external agents [13,14,15]. Any compound must overcome this asymmetric bilayer, composed of lipopolysaccharides (LPS) and phospholipids, to reach its target [15,16]. Three routes are available to overcome the OM (Figure 1): (a) direct diffusion, (b) active diffusion mediated by membrane receptors [17,18], and (c) facilitated diffusion by porins [17]. Porins represent a substantial fraction of the total OM proteins in *Enterobacteriaceae* (>10^5^ copies/cell) and thus they play a key role in compound permeation [15,17,18]. Molecules with a polar nature, such as fluoroquinolones, penicillin, cephalosporins, and carbapenems, use the porin route to enter the periplasmic space [19,20,21]. More hydrophobic molecules will directly diffuse, though slowly, through the bilayer [13]; while some specific molecules such as sugars or iron complexes have their specific receptors to be transported inside the cell [22,23]. Cell wall synthesis inhibitors (like β-lactams, Glycopeptides, Fosfomycin, Bacitracin, and Alafosfalin) and cell membrane disruptors (Lipopeptides) must only overcome this first barrier to reach their targets. It is worth noting that glycopeptides are only used to treat infections caused by Gram-positive bacteria due to their inability to overcome the OM in Gram-negative bacteria and hence reach their target. However, molecules targeting internal cell processes (Folate synthesis inhibitors, 30S, and 50S protein synthesis inhibitors, RNA synthesis inhibitors, DNA-dependent RNA polymerase inhibitors, and DNA gyrase inhibitors) must overcome the phospholipid-based inner membrane to reach their targets while avoiding specific enzymes and active efflux pumps.

Molecular uptake in Gram-negative bacteria can be a one-step or two-step process, depending on the location of the targets (Figure 1). Such a process is not controlled by the standard druglikeness rules such as the Lipinski rule of 5 and there is not a clear set of universal rules or physiochemical properties to assess molecular uptake prior to synthesis and test. Thus, most hits identified in HTS campaigns do not prosper to lead compounds due to poor molecular uptake or intracellular accumulation [11]. In the last four years, different groups have started to unveil a series of molecular descriptors/rules for predicting either permeation of molecules through the OM or accumulation of molecules in Gram-negative species. Our group unveiled the physical mechanism behind molecular uptake via porins from *Enterobacteriaceae* [24] and condensed this knowledge into a scoring function for compound permeability coefficient prediction and ranking [25]. With a different approach, the Hergenrother group analyzed a dataset of ~200 molecules and proposed some chemical modifications for successful intracellular accumulation. The authors conclude that the presence of a positively charged chemical group in the scaffold, i.e., primary amine, increases intracellular accumulation in *E. coli*. Nevertheless, from the 68 primary amines presents in the dataset only 36 of them accumulate in *E. coli*. Using a chemoinformatic approach the authors calculated 297 molecular descriptors to train a random forest classification algorithm to predict accumulation. From this analysis, the shape and flexibility of the molecule emerged as a determinant for molecules with a primary amine to overcome the OM and accumulate in *E. coli* [24], summarized in the eNTRY rules [26].

Other microbiological assays, such as bacterial growth inhibition in *E. coli*, have been combined with deep neural networks to find molecules with bactericidal activity against a wide phylogenetic spectrum of pathogens [27], but no general rules for molecular design or optimization were provided. Other machine learning approaches also used a random forest classification algorithm to identify the molecular properties selected by active efflux and the OM barrier. They found that antibiotic activity in *P. aeruginosa* was better classified by electrostatic and surface area properties, whereas topology, physical properties, and atom or bond counts capture best the behavior in *E. coli* [28]. This is not surprising as the porin composition of *E. coli* and *P. aeruginosa* are different, the former has different general porins that allow the passive diffusion of polar molecules while the latter is known for only having specific channels that are narrower and which exhibit specific motifs for substrate recognition [29].

With the aim to find the molecular determinants for predicting intracellular accumulation in *E. coli*, we considered here the above database for which accumulation data are available [26] and we calculated the dynamical molecular descriptors relevant for molecular permeability through porins. We evaluated the relevance of the physical descriptors behind successful permeation through porins in intracellular accumulation, as well as the correlation of the two processes. We finally put forward a complementary design strategy to the eNTRY rules that can be applied to compounds with intracellular targets but also periplasmic ones.

## 2. Results and Discussion

### 2.1. Structure Dependencies Found in the Whole-Cell Accumulation Data

**Figure 2:**
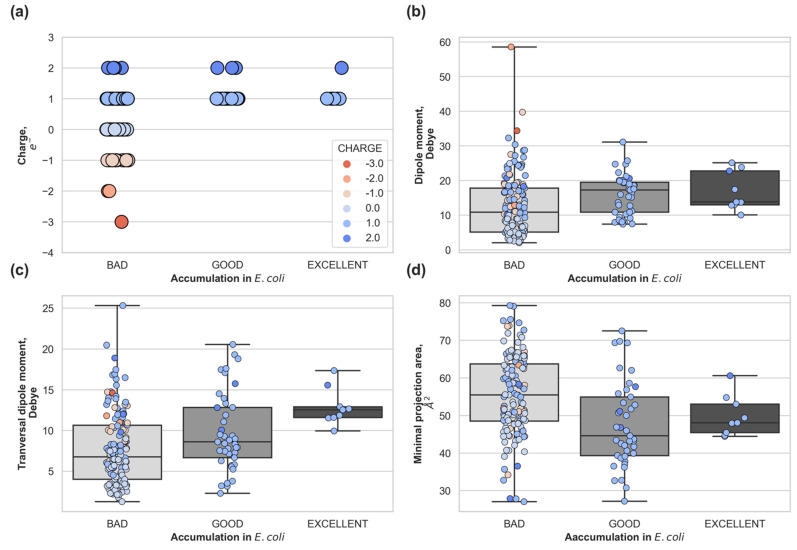
Whole-cell accumulation in *E. coli* versus molecular descriptors for the compounds with non-negligible accumulation: (**a**) total charge of the compound, (**b**) total dipole moment, (**c**) transversal dipole moment and (**d**) minimal projection area. Compounds are grouped as: Low Accumulators (<250 nmol per 10^12^ CFUs), Accumulators (<550 nmol per 10^12^ CFUs), Good Accumulators (>550 nmol per 10^12^ CFUs), and Excellent Accumulators (>1000 nmol per 10^12^ CFUs).

To establish the relevance of the physical descriptors controlling molecular uptake through porins [25] in intracellular accumulation, we calculated the net charge, minimal projection area, total and transversal dipole moment for all compounds measured in [26]. These physical descriptors arise from the description of the interactions between the molecule and the porin during the permeation process. In Figure 2, we depicted the distribution of descriptors, with compounds grouped by different experimental accumulation levels in *E. coli*: bad accumulators (<250 nmol per 10^12^ CFUs), good accumulators (>550 nmol per 10^12^ CFUs), and excellent accumulators (>1000 nmol per 10^12^ CFUs). Molecules that are electrostatically neutral (light blue) or negatively charged (pink, orange, red) are bad accumulators in *E. coli* (Figure 2 upper-left panel), which correlates with the preference for cations of the major porins OmpF/OmpC [30] in *E. coli*. Cation selective porins have an internal negative electrostatic potential and therefore, neutral, and negative molecules are expected to be disfavored during permeation. However, positively charged molecules (different shades of blues) are not always good accumulators, as it can be clearly observed in the number of data points falling into the bad accumulators category (Figure 2 upper left panel) with a net charge equal to +1 and +2. In the upper right panel of Figure 2, we can see that neutral molecules (light blue) present in the dataset have a very low total dipole moment (<10 Debye). *Good* and excellent accumulators are positively charged and have a total dipole moment bigger than neutral molecules (>10 Debye). Both the total dipole moment and the transversal dipole moment (Figure 2) show a trend in accumulation, the bigger the total/transversal dipole moment the better the accumulation. This is not the case for the minimal projection area (Figure 2 lower right panel), smaller molecules do not necessarily accumulate better, and excellent accumulators are, on average, bigger than good accumulators.

### 2.2. Predicting the Permeability Coefficient through Bacterial Porins

**Figure 3:**
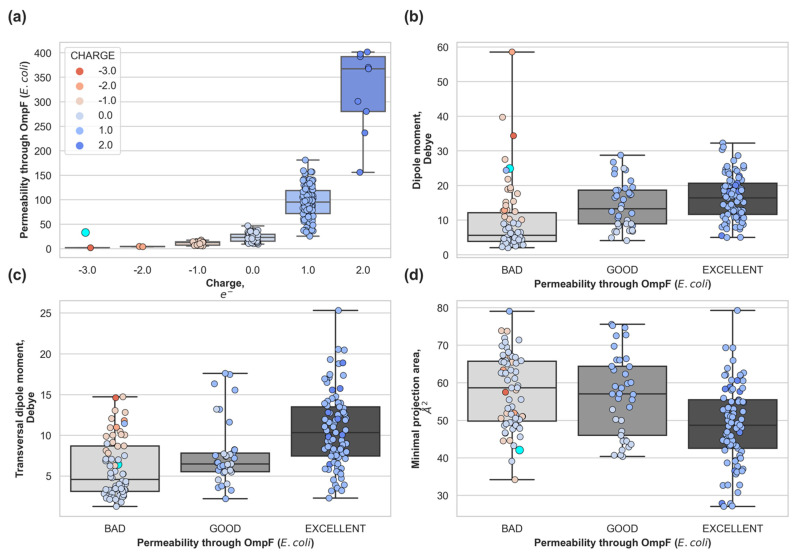
(**a**) Predicted permeability distribution according to the total charge of the compound. Molecular descriptors: (**b**) total dipole moment, (**c**) transversal dipole moment and (**d**) minimal projection area; for compounds with non-negligible accumulation grouped by predicted permeability through the major OmpF of *E. coli*. Very bad permeability (<30%), poor permeability (<50%), good permeability (70%), and excellent permeability (>70%). Percentages are relative to the measured [25] permeability coefficient of glycine through OmpF. Compound 183 (zwitterionic) is highlighted as a cyan dot with bigger marker size.

In Figure 3 we categorized the dataset in terms of the predicted permeability coefficient through OmpF (see Methods section) to analyze the distribution of molecular descriptors. Compounds were grouped according to the calculated permeability coefficient: *bad permeability* < 30%, *good permeability* < 70%, and *excellent* > 70%. We observed that the permeability coefficient increases exponentially with positive charge (Figure 3 upper left panel). As previously mentioned, OmpF is a cation selective pore and the negative electrostatic potential inside the pore favors positive molecules [25]. It is interesting to note that there is only one zwitterionic compound in the dataset (compound **183**, highlighted in cyan in Figure 3). Zwitterionic molecules are considered good permeating molecules in the literature [24,25,31], but this compound is a bad accumulator. This compound is predicted to have a permeability coefficient of 33%, which matches with its experimental poor accumulation. This is because although it is zwitterionic and its minimal cross-section is low (42 Å^2^), it is very rigid and with low transversal dipole moment (6.4 Debye).

The other three molecular descriptors show wide distributions for compounds exhibiting the same permeability level. The permeability coefficient depends on the delicate balance among the pore-molecule size distributions [32] (steric barrier) and the electrostatic interactions [24,25,33] that act as a barrier modulator. Hence, one molecular descriptor alone is not able to provide a good prediction.

### 2.3. Whole-Cell Accumulation versus the Permeability Coefficient through Bacterial Porins

**Figure 4:**
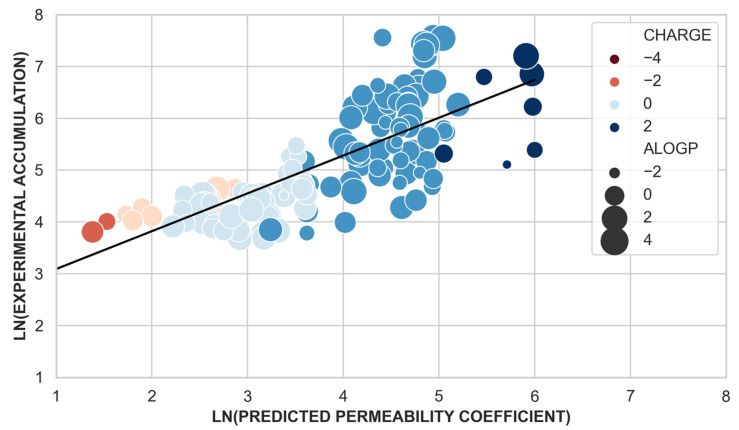
Natural logarithm of the experimental accumulation values (y-axis) versus the natural logarithm of the predicted permeability coefficient through OmpF. Data points are colored according to their charge and the point size relates to their hydrophobicity (alogP values). The linear regression model is depicted in black.

To estimate the importance of permeation via porins in compound accumulation, we evaluated the correlation between the experimental whole-cell accumulation data and the predicted compound permeability coefficient calculated with the scoring function [25]. Using a linear regression model, we obtained a correlation coefficient of R = 0.74 (*p*-value < 0.05, 137 data points, regression plots provided in Figure S3) (Figure 4). Despite the skewness of the dataset, only ~13% (24 molecules) of the total dataset showed high experimental accumulation (>550 nmol per 10^12^ CFUs) in *E. coli* [26], the ability of the scoring function to predict different degrees of permeability/accumulation is remarkable. It is worth noting that the scoring function was parametrized using a set of few clinically relevant antibiotics with good permeation. Although the present dataset comes from a different scaffold, our model is able to predict different permeability levels for them.

Taking a closer look at the data, molecules with low accumulation values in *E. coli* (bad accumulators) and with a predicted low permeability through OmpF (Supplementary Figure S1) are neutral or negatively charged but molecule number 150 (Table S1, Figure S2). This molecule despite being positively charged has low permeability and low cell accumulation due to its large average minimal projection area and low fluctuations (72 Å^2^ ± 1.8 Å^2^): it is big and rigid and its translocation through porins is unlikely. Molecules with high predicted permeability but negligible experimental accumulation are small (average minimal projection area of 49 Å^2^), polar (average alogP value 0.8), and positively charged (Table S1, Figure S2). These molecules might be able to permeate through porins but they are undetectable by the cellular accumulation assay either: (i) due to binding in the periplasmic space, as it has been reported for ampicillin [26], or (ii) due to efflux [34] out from the cell. New promising techniques allowing assessing the accumulation of compounds in the different compartments of the cell [35], will help to understand better why these compounds do not accumulate in bacteria.

**Figure 5:**
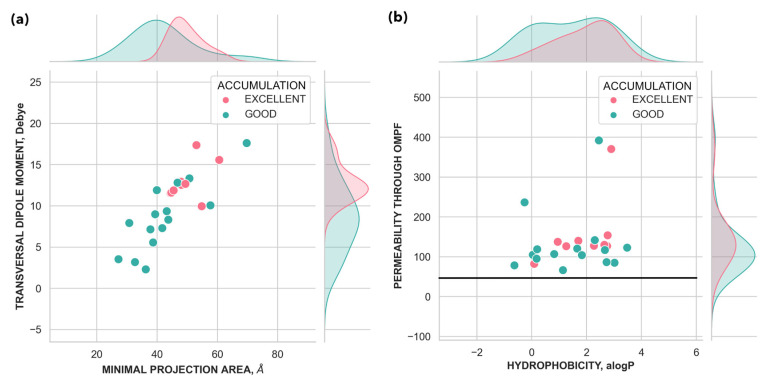
Molecular descriptors distributions for good/excellent accumulators: (**a**) compound minimal projection area and the transversal component of the total dipole moment and (**b**) permeability through OmpF versus hydrophobicity. Data points are colored according to good (green) or excellent accumulation values (magenta).

For molecules with excellent accumulation values and predicted to have *good* or *excellent* permeability (above 70% of permeability), two main conclusions can be stated: (i) the transversal dipole moment of excellent accumulators is always larger than 10 Debye (Figure 5a); (ii) good or excellent accumulators are not the molecules with the smallest cross-section area but in the range 40 and 50 Å^2^. Although hydrophobicity as a molecular descriptor alone does not correlate with accumulation [26], molecules with good/excellent accumulation are not molecules with a strong hydrophobic character (alogP < 2.9), as high hydrophobicity makes them more likely to be subject to efflux [34]. However, ‘good accumulators’ are not very hydrophilic (alogP > −1) although they need to diffuse through the inner membrane [36,37] to effectively accumulate in the cytoplasm of the bacterial cell.

## 3. Methods

### 3.1. Dataset and Molecular Descriptors Calculation for the Scoring Function

All compounds used in this work were obtained from the Supporting Information of Richter et al. [26]. We extract the smiles for all 189 different compounds provided in the public dataset of Richter et al. [26]. After generating the 3D structure of each compound using MARVIN [38] we performed a structural optimization followed by a semi-empirical parametrization using antechamber [39,40]. Each compound was solvated in a TIP3P3 [41] water box of 20 Å and a molecular dynamics simulation of 100 ns was conducted with ACEMD [42] to calculate the distributions of the molecular dipole and minimal projection area. Alog*p* values were calculated using the alogps 2.1 server [43].

We calculated the permeability coefficient of each compound in the dataset through OmpF using a previously published scoring function [25]:

(1) $$P_{OmpF}=\alpha \cdot U_{steric}+\beta \cdot Q_{molecule}\cdot V_{pore}+\gamma \cdot D_{molecule}\cdot E_{pore}+\delta$$

where $U_{steric}$ is the steric term due to the size-exclusion of hourglass-shaped porins [25,32]; $Q_{molecule}$ is the net charge of the molecule, $V_{pore}\;$ is the electrostatic potential of the pore, $D_{molecule}$ is the transversal component of the dipole moment of the molecule and $E_{pore}$ is the characteristic transversal electric field of the pore. It must be noted that the intensity of the internal electric field of general porins in the diffusion axis direction is negligible and, hence only the transversal component is considered [24,25,33,44]. To calculate the steric contribution ($U_{steric}$) the size of the molecule and its flexibility are calculated via the molecular minimal projection area (MPA) and its standard deviation. Due to the characteristic hourglass shape of porins, a more suitable molecular descriptor for the size is the MPA of a molecule than its mass, as a very long polymer will translocate through a pore despite with a very high molecular weight, if it has a small MPA [45]. Furthermore, the flexibility of the molecule is important because we noted that the average size of molecules is larger than the average size of the general porins constriction region [32]. Thus, the fluctuations of the MPA and the porins size must be considered. In calculating the permeability of molecules in the dataset, we used the same coefficients (α, β, γ, and δ) that were trained in [25].

### 3.2. Data Manipulation

To calculate the correlation in Figure 4 we discarded molecules with very low experimental accumulation values. The cutoff for experimental accumulation value was selected according to the accumulation of ampicillin in the original work [26], 45 nmol per 10^12^ CFUs. Ampicillin is rapidly covalently appended to penicillin-binding proteins, preventing measurement by LC-MS/MS, and therefore all molecules with experimental accumulation lower than 45 nmol per 10^12^ CFUs were discarded (Figure S1, 34 datapoints/molecules). Non-polar molecules were also discarded from the dataset as they do not penetrate via porins (alogP > 2.9). By discarding molecules with negligible experimental accumulation (34 data points) and non-polar compounds (17 data points), we reduced the original dataset published from 189 to 137 molecules. This subset was considered for the linear regression analysis only, Figure 4.

## 4. Conclusions

In this work, we showed that the molecular descriptors related to the pore-molecule interactions, useful to predict permeation of molecules through porins, are also key to describe the accumulation of molecules in Gram-negative bacteria. Our model to predict molecular permeability through an interaction-based scoring function can be extended to predict also accumulation, easier to measure in experiments. Furthermore, our scoring function is able to discriminate different levels of accumulation, without any re-training of the parameters. Moreover, because the model incorporates information about porins, it is readily extendable from *E. coli* to multiple species with different OM compositions, in terms of differently expressed porins.

The analysis of the dataset in terms of permeability through porins offers valuable insights into this complex macroscopic process: (i) the correlation between the permeability coefficient through porins and experimental accumulation (0.74) highlights general porins as the main permeation pathway to cross the OM. (ii) Good/excellent accumulators are polar and have a high permeability coefficient through porins. (iii) Apart from being positively charged (net charge), the charge distribution of excellent accumulators maximizes the transversal component of their dipole moment with respect to its main axis of inertia. Therefore, the only way to operate on permeation to obtain good accumulation is to optimize the position of charged groups in the molecular scaffold. (iv) Small compounds are predicted to have good/excellent permeation, but excellent accumulators are bigger than good accumulators.

## Data Availability

The data that support the findings of this study are available from the corresponding author, S.A.G., upon reasonable request.

## Supplementary Materials

The following are available online at https://www.mdpi.com/article/10.3390/antibiotics10060635/s1, Figure S1: Molecules with negligible experimental cell accumulation, Figure S2: Molecular descriptors of discarded molecules with negligible accumulation, Figure S3: Regression plots for the linear regression model presented in Figure 4, Table S1: Molecular descriptors of discarded molecules with negligible accumulation.
