# Supplementary material for: The Influence of Permeability through Bacterial Porins in Whole-Cell Compound Accumulation
Source: Antibiotics (Basel). 2021 May 26;10(6):635. doi: 10.3390/antibiotics10060635 (PMC8226570; doi:10.3390/antibiotics10060635)
Supplement: Supplementary file 1 [file antibiotics-10-00635-s001.zip › antibiotics-1211707-supplementary.pdf]

antibiotics

MDPI

Supplementary materials :The influence of permeability through bacterial porins in whole cell compound accumulation

Silvia Acosta-Gutiérrez [1,2,3,4,*], Igor V. Bodrenko [5] and Matteo Ceccarelli [5,6,7].

[1.] Physical Chemistry Chemical Physics Division Department of Chemistry, University College London, London, UK.
[2.] Institute of Structural and Molecular Biology,University College London, London, UK.
[3.] Institute for the Physics of Living Systems,University College London, London, UK
[4.] EPSRC/JEOL Centre for Liquid Phase Electron Microscopy, University College London, London, UK
[5.] IOM/CNR, Sezione di Cagliari, Cittadella Universitaria di Monserrato, 09042 Monserrato, Italy
[6] Department of Physics, University of Cagliari, 09042 Monserrato, Italy
[7] CNR/IOM, Cittadella Universitaria di Monserrato, 09042 Monserrato, Italy

Table S1. Molecular descriptors of discarded molecules with negligible accumulation. Predicted permeability is considered very low (<30%), low (<50%), moderate (<70%), high (<100%) and very high (>110%).

Molecule	Min. Area (Å²)	Min. Area Fluctuations (Å²)	Total Dipole Moment (Debye)	Transversal Dipole Moment (Debye)	Charge (e)	AlogP	Accumulation (nmol per 10^{12} CFUs)	Predicted permeability
8	49.2	4.2	24.2	10.3	1.0	1.69	7.0 ± 1.0	Very High
10	55.3	0.97	12.9	10.7	1.0	-1.26	30.0 ± 1.0	High
11	50.3	3.9	23.8	9.4	1.0	-1.64	30.0 ± 1.0	High
20	27.8	0.4	12.8	3.9	1.0	-1.78	0.0 ± 0.0	High
29	43.4	0.4	15.9	5.9	1.0	5.32	28.0 ± 6.0	High
39	27.9	0.2	5.5	5.4	2.0	-2.1	20.0 ± 2.0	Very High
40	27.06	0.36	12.76	3.91	1.0	-1.78	0.0 ± 0.00	High
52	62.3	3.3	11.9	5.9	1.0	2.12	16.0 ± 4.0	Moderate
63	66.1	0.56	17.8	13.2	1.0	1.12	3.0 ± 0.0	Moderate
64	63.9	2.14	17	14.8	1.0	4.17	1.0 ± 0.0	High
121	48.5	0.1	5.0	4.5	1.0	0.47	18.0 ± 7.0	High
148	58.0	2.2	24.1	16.9	1.0	5.33	38.0 ± 12.0	Very High
149	36.5	1.8	10.1	9.8	2.0	-2.05	23.0 ± 0.0	Very High
151	66.0	1.3	25.5	25.3	1.0	3.18	17.0 ± 7.0	Very High
152	58.6	1.5	12.6	11.9	1.0	2.53	16.0 ± 6.0	High
188	58.4	1.4	14.6	13.9	1.0	-1.13	5.0 ± 0.1	High
189	50.9	0.9	12.2	7.5	1.0	0.8	0.0 ± 0.0	High
14	71.9	4.1	5.7	5.3	0.0	1.32	8.0 ± 1.0	Very Low
80	44.5	0.68	18.8	8.7	-1.0	5.28	34.0 ± 5.0	Very low
81	73.7	3.4	19.3	9.9	-1.0	0.95	17.0 ± 0.0	Very low
82	66.8	2.6	14.2	10.1	-1.0	-0.26	17.0 ± 1.0	Very low
83	58.1	3.7	39.7	7.8	-1.0	1.87	17.0 ± 1.0	Very low
84	50.0	2.7	13.3	13.2	0.0	0.64	15.0 ± 10.0	Low
111	50.2	1.3	2.5	2.3	0.0	-1.06	33.0 ± 2.0	Very Low
112	62.6	3.4	3.3	2.7	0.0	-1.06	31.0 ± 3.0	Very Low
113	40.7	2.9	4.9	4.5	0.0	-1.06	29.0 ± 13.0	Very Low
114	65.0	1.1	4.2	3.8	0.0	0.34	0.0 ± 0.0	Very Low
115	65.5	0.8	4.2	3.8	0.0	-0.05	0.0 ± 0.0	Very Low
150	72.7	1.8	28.8	8.2	1.0	2.97	20.0 ± 5.0	Very Low
166	48.7	0.8	6.2	4.0	0.0	0.30	21.0 ± 1.0	Very Low
168	58.7	2.9	6.4	6.2	0.0	1.56	38.0 ± 5.0	Very Low

170	44.4	4.1	6.7	5.5	0.0	1.61	25.0 ± 0.0	**Very Low**
176	39.1	3.0	3.3	3.1	0.0	2.29	20.0 ± 0.0	**Very Low**
181	50.6	2.7	4.6	3.5	0.0	3.29	28.0 ± 12.0	**Very Low**

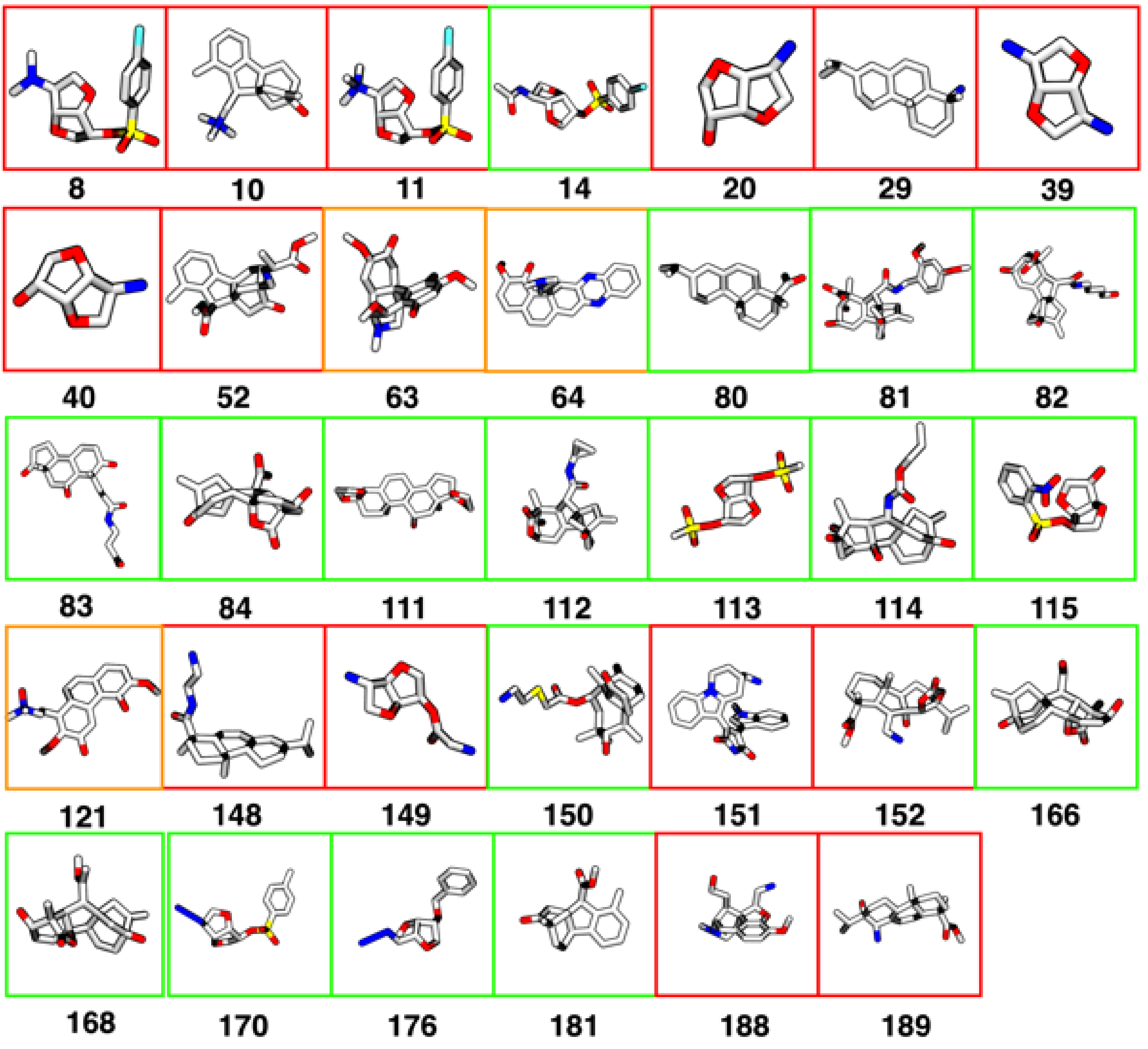

Figure S1. Molecules with negligible experimental cell accumulation. Molecules predicted to have a high or very high permeability through porins are highlighted in red (false positives) while molecules predicted to have a low or very low permeability through porins are highlighted in green (true positives) and molecules with medium or moderate permeability are highlighted in orange.

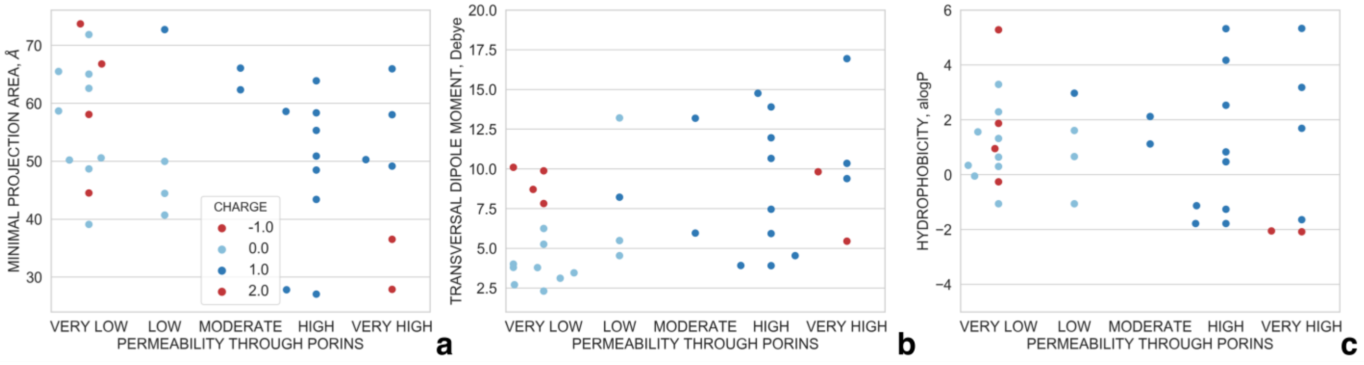

Figure S2. Molecular descriptors of discarded molecules with negligible accumulation. Predicted permeability is considered very low (<20%), low (<50%), moderate (<70%), high (>70%) and very high (>120%).

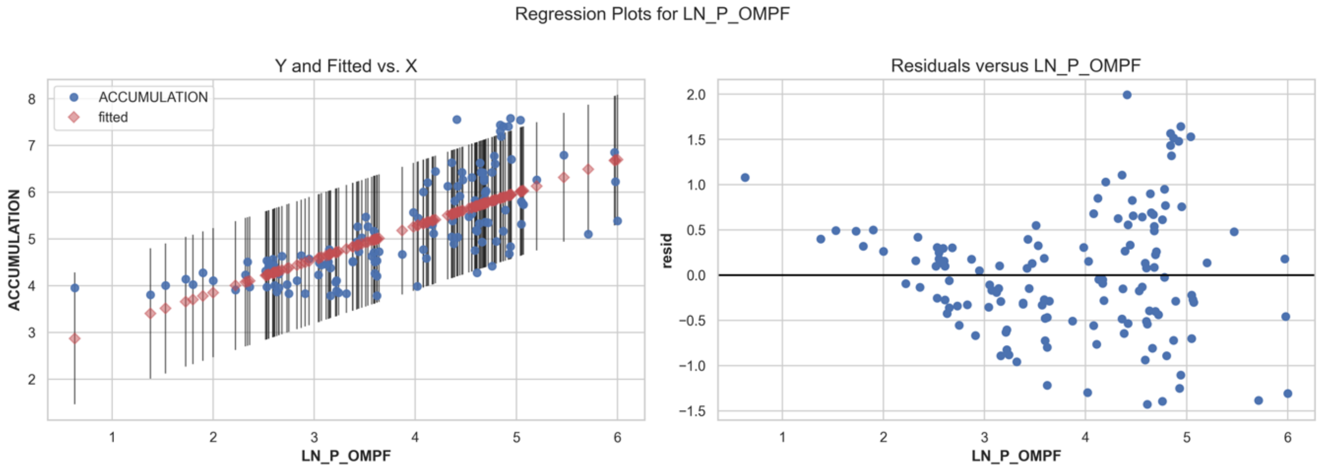

Figure S3. Regression plots for the linear regression model presented in Figure 4. Left: Experimental Accumulation [26] versus fitted value with linear model presented in Figure 4. Residuals plot.
